# The Mechanosensitive Ion Channel Piezo1 Regulates Chondrocyte Homeostasis Through the PI3K/AKT/mTORC1 Pathway in Osteoarthritis

**DOI:** 10.1111/jcmm.70734

**Published:** 2025-07-31

**Authors:** Yuanyuan Han, Fangyan Cheng, Xinyan Li, Jiahao Yu, Guimiao Li, Wei Chen, Jiaxue Zhang, Chen Feng, Yingze Zhang, Juan Wang

**Affiliations:** ^1^ Orthopaedic Research Institution of Hebei Province Shijiazhuang China; ^2^ NHC Key Laboratory of Intelligent Orthopaedic Equipment Hebei Medical University Third Hospital Shijiazhuang China; ^3^ Medical General Laboratory Hebei Medical University Third Hospital Shijiazhuang China; ^4^ Department of Orthopaedic Surgery Hebei Medical University Third Hospital Shijiazhuang China; ^5^ Key Laboratory of Biomechanics of Hebei Province Shijiazhuang China; ^6^ Hebei Orthopedic Clinical Research Center Hebei Medical University Third Hospital Shijiazhuang China; ^7^ Department of Joint Surgery Hebei Medical University Third Hospital Shijiazhuang China

**Keywords:** chondrocyte homeostasis, mechanical stress, mTORC1, osteoarthritis, PI3K/AKT, Piezo1

## Abstract

Osteoarthritis (OA) is a highly prevalent chronic joint disease with complicated pathogenesis, causing pain and dysfunction. The mechanosensitive ion channel Piezo1 is an important mechanoreceptor on chondrocytes and may be involved in the process of OA. However, its role and mechanism remain elusive. This study investigated the role and possible mechanisms of Piezo1 in OA induced by excessive mechanical stress. Piezo1 expression levels were markedly up‐regulated in the lesioned region of the medial tibial cartilage in OA rats subjected to destabilisation of the medial meniscus (DMM) surgery. Excessive mechanical stress increases Piezo1 in chondrocytes, which leads to a reduction in collagen II (COL2) and aggrecan (ACAN). In addition, activation of Piezo1 channels by the specific agonist Yoda1 reduces chondrocyte anabolism and promotes catabolism. Molecularly, activation or inhibition of Piezo1 modulates the activity of the PI3K/AKT/mTORC1 pathway. Inhibition of the PI3K/AKT pathway or mTORC1 alleviated the Yoda1‐induced imbalance of chondrocyte homeostasis to varying degrees. Collectively, these findings imply that Piezo1 in chondrocytes may respond to excessive mechanical forces and promote extracellular matrix degradation through the PI3K/AKT/mTORC1 pathway, thereby driving OA progression.

## 1 Introduction

OA is a whole‐joint disease primarily characterised by articular cartilage degeneration, accompanied by osteophyte formation, synovial inflammation, and involving meniscal degeneration, subchondral bone remodelling, as well as pathological alterations in the infrapatellar fat pad [1, 2, 3]. It is also a major factor in chronic pain and disability among older adults [4]. The incidence of OA is continuously rising as the population ages and obesity increases, but effective treatments are lacking [5]. It is therefore crucial to understand how OA develops and to identify potential targets. The causative factors of OA are complicated, including factors such as obesity, age, gender and trauma. Mechanical overload caused by obesity, trauma or joint instability is a major trigger for OA and results in irreversible damage to cartilage [6, 7]. However, the specific mechanism is not yet fully understood.

Articular cartilage consists of chondrocytes and extracellular matrix. Chondrocytes, as the only cell type of articular cartilage, respond to mechanical stimuli in the microenvironment, thereby controlling the synthesis and degradation of extracellular matrix components [8, 9]. Under physiological levels of mechanical loading, chondrocytes maintain homeostasis with a balance between anabolism and catabolism, and the extracellular matrix of cartilage tissue is intact [10]. Under supra‐physiological levels of mechanical loading, however, chondrocytes exhibit hypertrophic and catabolic phenotypes, with aberrant production of the pro‐catabolic enzymes MMPs (e.g., MMP3, MMP13) and ADAMTS4/5, which degrade the extracellular matrix components COL2 and proteoglycans, leading to chondrogenic degeneration and facilitating OA progression [11, 12, 13]. Critically, abnormal mechanical stress not only induces metabolic imbalance but also alters the intrinsic biomechanical properties of chondrocytes—including increased cellular stiffness, cytoskeletal reorganisation, and pericellular matrix remodelling. These changes further disrupt mechanotransduction pathways, creating a vicious cycle that exacerbates OA pathogenesis [14].

The Piezo family is a category of mechanosensitive calcium channel proteins, encompassing two distinct members, namely, Piezo1 and Piezo2, which were first discovered and reported by Coste et al. in 2010, attracting widespread attention in the scientific community [15]. Subsequent studies have shown that they are key molecules in the perception and conduction of mechanical forces in many types of cells, play important roles in different tissues and organs, and are closely related to human health and disease [16, 17]. Piezo proteins, especially Piezo1, have also been reported to play important mechanotransduction roles in articular cartilage, which is highly expressed on chondrocytes and activates calcium signalling pathways in response to supraphysiological levels of mechanical force [18, 19, 20]. The activation of Piezo1 is intricately linked to apoptosis, ferroptosis, senescence and inflammatory signalling in chondrocytes and may mediate the biomechanical adaptation of chondrocytes by regulating cytoskeletal organisation and cellular stiffness, ultimately impacting the pathogenesis and progression of OA [21, 22, 23]. Nevertheless, the precise involvement of Piezo1 channels in OA induced by abnormal mechanical stress remains elusive, and the underlying molecular mechanisms remain largely unclear.

In this investigation, we used DMM surgery to simulate OA caused by abnormal mechanical loading and observed up‐regulation of PIEZO1 in cartilage lesions. On the basis of these findings, this study aims to: 1) elucidate whether Piezo1 activation directly drives excessive mechanical stress‐induced chondrocyte metabolic dysfunction; 2) determine the role of PI3K/AKT/mTORC1 signalling in Piezo1‐mediated cartilage degradation.

## 2 Materials and Methods

### 2.1 Animals

Female Sprague–Dawley (SD) rats were acquired from Charles River Laboratories (Beijing, China). The rats were housed in IVCs under controlled conditions (22°C ± 1°C, 55% ± 5% humidity, 12 h light/dark cycle) with environmental enrichment and free access to food and water. Primary articular chondrocytes were isolated from female rats aged 4 weeks (body weight 80 ± 10 g, *n* = 6), whereas female rats aged 8 weeks (body weights: 200 ± 15 g, *n* = 20) were used for in vivo experiments. All experimental procedures were approved by the Laboratory Animal Ethics and Welfare Committee of Hebei Medical University (IACUC‐Hebmu‐2022044).

### 2.2 DMM‐Induced Rat OA Models

8‐week‐old female rats were acclimatised for at least 7 days prior to experiments. A rat OA model was established by DMM surgery. The rats were randomly assigned into two groups (*n* = 10 each) by body weight using GraphPad Prism v9: DMM surgery (bilateral knee) and sham surgery (arthrotomy only). Subsequently, the animals were further divided into 2 observation time points: 2 weeks and 8 weeks postoperatively, with *n* = 5 per subgroup as determined by power analysis (α = 0.05, power = 0.8, effect size = 1.8 on the basis of pilot data; G*Power 3.1).

Detailed descriptions of the specific surgical procedures have been previously provided [24]. Briefly, after anaesthetising the rats with isoflurane (5% induction, 1.5%–2% maintenance, O2 flow rate 1 L/min), the skin and joint capsule were aseptically incised on the medial parapatellar side of the knee. Then, the medial meniscotibial ligament was severed in order to establish a DMM model. In contrast, the sham‐operated group had only incised the skin and joint capsule. Rats were monitored daily (days 1–3) then weekly. Humane endpoints: > 20% weight loss or lameness score ≥ 3. There were no exclusions (final *n* = 5/subgroup).

### 2.3 Histological Assessment and Immunofluorescence

Following euthanasia by CO_2_ inhalation (30% chamber volume/min) with cervical dislocation confirmation, the whole knee joints of rats were taken, fixed in 4% paraformaldehyde for 24 h. Then, a modified Perenyi's decalcifying fluid was employed to facilitate decalcification. After decalcification, samples were paraffin‐embedded and cut into 3.5 μm coronal sections (Leica, USA). From these sections, one section was selected every 200 μm and subjected to staining with Safranin‐O/Fast Green. The overall structure of the cartilage, chondrocytes, matrix staining and tidal lines were scored by two independent pathologists using a modified Mankin score system to assess the severity of OA (Mild OA: 0–5 points, Moderate OA: 6–10 points, Severe OA: 11–14 points) [25].

For immunofluorescence analysis, the sections underwent overnight alkaline repair at 55°C. Subsequently, they were blocked with 5% donkey serum for 1 h, followed by incubation with anti‐Piezo1 primary antibody (1:100, 15939–1‐AP, proteintech) overnight at 4°C. Then, they were further treated with donkey anti‐Rabbit Alexa Fluor 488 conjugated secondary antibody (A‐21206, Thermo Fisher Scientific, USA) in the absence of light for 1 h at room temperature, at a dilution of 1:500. To visualise cell nuclei, an antifade mounting medium containing DAPI (H‐1200, Vector Labs, USA) was used. Sections were imaged using an Olympus IX53 fluorescence microscope. Positive cells were quantified using ImageJ software. The percentage of Piezo1 positive cells was calculated using the formula: (Number of Piezo1 positive cells ÷ Total cell count) × 100%.

### 2.4 Extraction and Culture of Rat Primary Chondrocytes

Primary rat chondrocytes were extracted from knee cartilage of female 4 weeks old SD rats as indicated previously [26]. Firstly, the rats were euthanised and subjected to aseptic sterilisation by immersion in 75% alcohol for 15 min. Under sterile conditions, the knee cartilage tissue of the rats was carefully dissected, ensuring the removal of surrounding connective tissues such as synovium and tendon. The articular cartilage was then sliced into 1–3 mm fragments using a scalpel. These slices were subsequently washed 3 times with PBS before undergoing overnight digestion using 0.2% collagenase‐2 (v900892, Sigma) in a controlled cell incubator. On the following day, the chondrocytes were harvested through centrifugation and then suspended in L‐DMEM (Dulbecco's modified Eagle's medium) with 10% fetal bovine serum and 1% penicillin–streptomycin (100 U/mL penicillin +100 μg/mL streptomycin). The cells were grown in an incubator with 5% CO_2_ at 37°C, and the medium was refreshed every other day. Chondrocytes within the first three passages (P0‐P2) were utilised for subsequent experiments.

### 2.5 Quantitative Real‐Time PCR (qRT‐PCR)

Total RNA from rat chondrocytes was extracted employing a High Purity RNA Extraction Kit (ZS‐M11005, zsgentech, China) on the basis of the instructions. Subsequently, the RNA underwent conversion into cDNA using the PrimeScript RT reagent Kit with gDNA Eraser (RR047A, Takara, Japan). For qRT‐PCR experiments, an ABI 7500 Real‐Time PCR System (Applied Biosystems, USA) was utilised, along with TB Green Premix Ex Taq II (RR820A, Takara, Japan). The PCR cycling conditions were as follows: initial denaturation at 95°C for 30 s, followed by 40 cycles of 95°C for 5 s and 60°C for 30 s. Melting curve analysis was performed with 95°C for 15 s, 60°C for 1 min, and 95°C for 15 s (ramp rate: 0.3°C/s). The mRNA expression was normalised with GAPDH as the reference gene. Each sample was subjected to analysis in triplicate, and the relative mRNA expression level was computed using the fold change of the *2* ^*−ΔΔCT*^ method. Table 1 lists the primer sequences used for qPCR.

**TABLE 1:** Primer sequences for qRT‐PCR in rat.

Gene	Forward primer (5′‐3′)	Reserve primer (5′‐3′)
*Piezo1*	CGCAACCTCACGGGCTTC	GGGCCTCGCTCACTGTATCC
*Col2a1*	TGGAGAGAAAGGCGAACC	CAGGCAGACCAACAATGC
*Acan*	CTAGCTGCTTAGCAGGGATAACG	GATGACCCGCAGAGTCACAAAG
*Gapdh*	ATGGCTACAGCAACAGGGT	TTATGGGGTCTGGGATGG

### 2.6 siRNA Transfection

*Piezo1*‐targeted siRNA and negative control siRNA (NC siRNA) were synthesised by Sangon Biotech (Shanghai, China) and handled in accordance with the instructions. Briefly, the chondrocytes were cultured to 40%–60% density, and Piezo1 siRNA or NC siRNA was transfected with RNA transmate (#E607402, Sangon Biotech, China) at 10 nM for 48 h. The efficacy of silencing was confirmed through qRT‐PCR and western blot analysis. In order to identify the most effective siRNA sequence, three different sequences were assessed, ultimately leading to the selection of the optimal siRNA for subsequent cellular experiments. The sequences of the *Piezo1* siRNA are listed in Table S1.

### 2.7 Cyclic Tension Stress (CTS)

Rat primary chondrocytes were inoculated at a density of 2 × 10^5^ cells/well in six‐well flexible Flexcell culture plates (Flexcell International Corporation, USA) encapsulated with type I collagen. The cells were then cultured for 2–4 days until reaching 70%–90% confluence. Subsequently, the chondrocytes were subjected to mechanical strain using the FX‐5000 tension system. The cyclic tension stress was applied at 17% elongation, using a square waveform at a frequency of 0.5 Hz for 6, 12, and 24 h. These parameters were chosen to simulate the excessive mechanical stress experienced by chondrocytes in the destabilised medial meniscus (DMM) model (mimic pathological mechanical loading conditions associated with OA). The control cells were cultured under identical experimental conditions but were not exposed to mechanical stress. Finally, RNA or proteins were harvested from the cells for further investigations.

### 2.8 Calcium Imaging

To determine the levels of calcium ions within chondrocytes, primary chondrocytes were inoculated into 35 mm glass‐bottom confocal dishes (Beyotime Biotechnology, China) and cultivated in L‐DMEM until reaching 60% confluence. Cells were exposed to 5 μM Fluo‐8 AM dye (#21082, *AAT Bioquest, USA)* for 45 min at 37°C and washed using Hank's buffer with 20 nM Hepes (HHBS) solution to remove excess probe. The fluorescence intensity of calcium ions was continuously monitored using a Two‐photon Laser Scanning Microscope (Olympus, Japan). Once the baseline reached a plateau, HHBS or 10 μM Yoda1 was meticulously introduced, and images were captured consecutively for a duration of 10 min at intervals of 3 s. The change in intracellular calcium concentration was calculated as (*F*_*x*_ *‐F*_*0*_)*/F*_*0,*_ *F*_*x*_ represents the absolute fluorescence intensity at a given time point, and *F*_*0*_ denotes the baseline fluorescence intensity.

### 2.9 RNAseq Analysis

Primary chondrocytes from each group (*n* = 3 biological replicates) were lysed in TRIzol reagent (Invitrogen, Carlsbad, CA, USA) and stored at −80°C. All samples were subsequently transported to Genergy Biotechnology (Shanghai, China) under strict cold‐chain conditions for RNA sequencing analysis. Following extraction, RNA quality was assessed by gel electrophoresis and with Qubit fluorometry (Thermo, Waltham, MA, USA). 1 μg of total RNA was used for library construction. Sequencing libraries were prepared using the TruSeq RNA sample preparation kit (Illumina, San Diego, CA, USA) and subjected to high‐throughput sequencing using the Illumina Novaseq6000 sequencing platform (150 bp paired‐end reads). Raw reads were processed with Skewer and FastQC (v0.11.2), then aligned to hg38 using STAR (v2.5.2b) and assembled with StringTie (v2.2.1). The DESeq2 software (v1.16.1) was employed to screen for differentially expressed genes (DGEs) between groups. The significance thresholds for differential gene expressions are *p* < 0.05 (unadjusted) and absolute fold change ≥ 2. Although we initially employed unadjusted *p* values to enhance detection sensitivity for biologically relevant genes in our small sample size study, we also calculated FDR‐adjusted *q* values for all genes to provide complementary statistical rigour. Gene Ontology (GO) enrichment was analysed by TopGO (v2.59.0), and pathway analysis was conducted using Reactome and KEGG databases. The raw sequencing data generated in this study have been deposited in the NCBI Sequence Read Archive (SRA) under accession number PRJNA1196021.

### 2.10 Pharmacological Inhibition

To investigate the role of the PI3K/AKT/mTOR pathway, chondrocytes were pretreated for 2 h with either 25 μM LY294002 (MedChemExpress, HY‐10108) to inhibit PI3K/AKT signalling or 100 nM rapamycin (MedChemExpress, HY‐10219) to suppress mTORC1 activity. Both inhibitors and controls used DMSO as a vehicle. After pretreatment, cells were exposed to DMSO or Yoda1 for 12 h before protein extraction for Western blot analysis.

### 2.11 Western Blotting

Chondrocytes were lysed using RIPA buffer containing protease inhibitors Cocktail (RP‐WA0102, Reportbio, China) and PMSF and incubated for 20 min to obtain total cellular protein. Protein concentration was determined with a BCA kit (Reportbio, China). Following the addition of sample buffer, total cellular protein was denatured at 95°C for 10 min. Equal amounts of protein (20 μg per lane) were loaded and separated on 4%–12% gradient sodium dodecyl sulfate‐polyacrylamide gel electrophoresis (SDS‐PAGE) and then electrotransferred to polyvinylidene difluoride (PVDF) membranes. After blocking with 5% nonfat milk for 1 h, the membranes were incubated overnight at 4°C with primary antibodies targeting Piezo1 (1:500, 15939–1‐AP, proteintech), GAPDH (1:1000, ET1601‐4, Huabio), COL2 (1:1000, GTX100829, Genetex), MMP13 (1:1000, 18165–1‐AP, proteintech), MMP3 (1:1000, ER1706‐77, Huabio), ACAN (1:1000, DF7561, affbiotech), ADAMTS5 (1:1000, ab41037, abcam), Phospho‐PI3K p85 alpha (Tyr607) (1:1000, AF3241, affbiotech), PI3K (1:1000, ET1608‐70, Huabio), Phospho‐Akt (Ser473) (1:1000, #4060, cell signalling technology), AKT (1:1000, ET1609‐47, Huabio), and mTOR pathway kit (1:1000, #9964, cell signalling technology). HRP‐labelled secondary antibody was applied, and signals were detected using a ChemiDoc imaging system (Bio‐Rad, USA). The protein bands were quantified with ImageJ, with GAPDH expression from each sample employed for protein normalisation.

### 2.12 Statistical Analysis

Data were statistically evaluated using GraphPad Prism 8.0 software (GraphPad Software, San Diego, CA, USA). Differences between the two groups were examined using an independent samples *t*‐test. Statistical differences were analysed using one‐way ANOVA followed by Tukey's post hoc test for multiple comparisons for 3 or more groups. Each group had at least three independent repetitions. Data were expressed as mean ± SD. Statistically significant differences were indicated by a *p* < 0.05.

## 3 Results

### 3.1 Up‐Regulation of PIEZO1 Expression in the Medial Cartilage Lesion Area of the Tibial Plateau in a Rat OA Model

**FIGURE 1:**
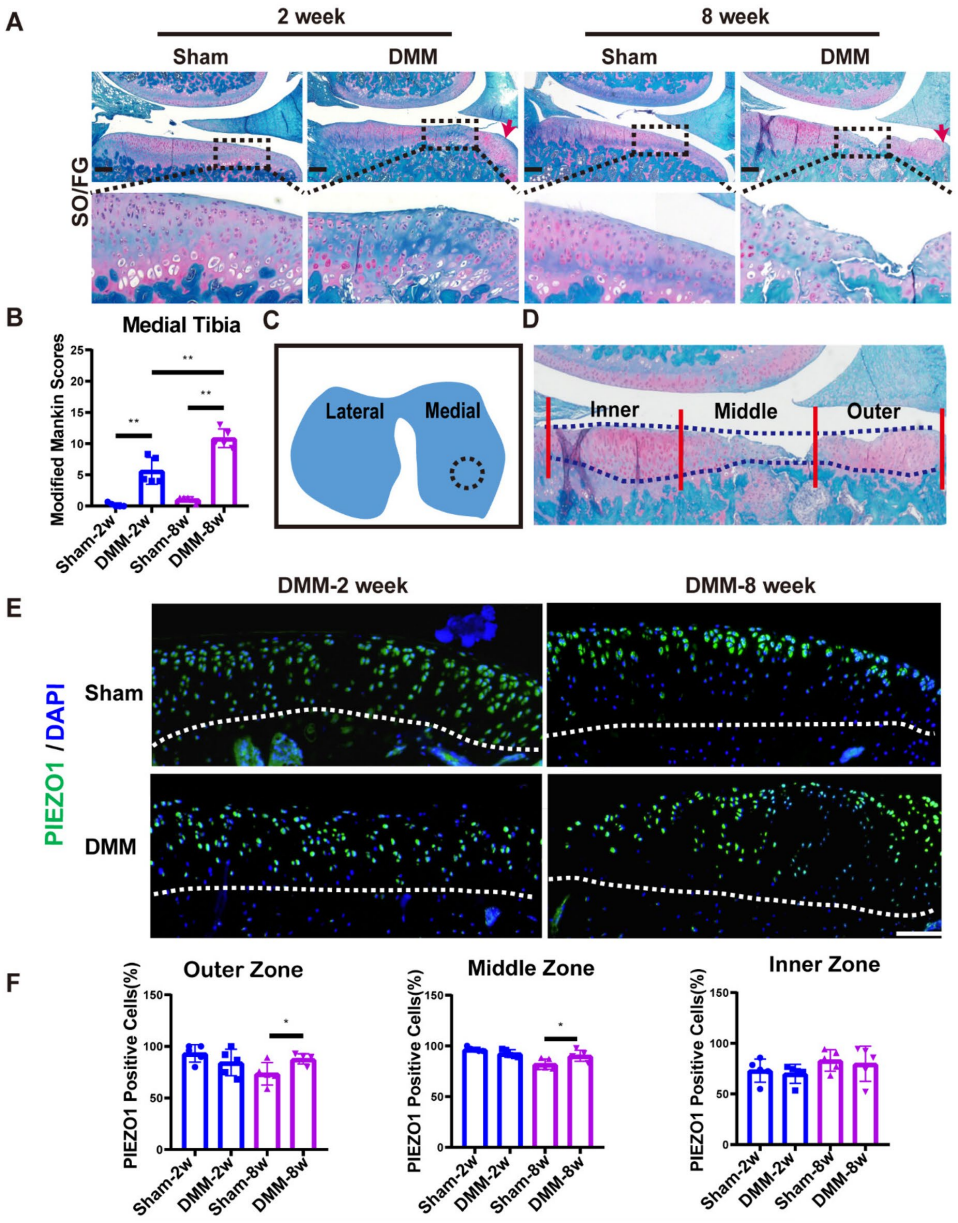
The expression of Piezo1 is elevated in cartilage lesions of rats with DMM‐induced OA. (A) Typical images of SO/FG staining of the medial tibial plateau cartilage at 2 and 8 weeks post sham or DMM operation. The red arrow indicates osteophytes. The scale bar is 200 μm. (B) The modified Mankin score at 2 and 8 weeks after sham or DMM surgery is presented (*n* = 5). (C) A schematic illustration depicts cartilage lesions in the medial tibial plateau after DMM surgery. The blue area indicates the coronal tibial plateau. The area surrounded by the dotted line is the central area of the cartilage lesion. (D) The medial tibial cartilage was separated into three equal‐width zones from the medial joint margin to the central ligament using a visual or pictorial measuring tape, in the order of the outer zone, middle zone, and inner zone. Three areas are delineated with red lines. The blue line traces the tidal line and the projected cartilage surface. (E) Typical images of PIEZO1 immunofluorescence staining in the region of cartilage lesion at 2‐ and 8‐weeks following sham or DMM, with white dashed lines indicating the tidemarks. Scale Bar, 100 μm. (F) Statistical graph of the percentage of PIEZO1‐positive cells in the outer zone, middle zone and inner zone at 2 and 8 weeks after sham or DMM (*n* = 5). The data were shown as mean ± SD. *p < 0.05, **p < 0.01.

8‐week‐old SD rats underwent bilateral knee DMM or sham surgery. Subsequently, rat knee joints were obtained at 2 and 8 weeks after surgery for Safranin‐O/Fast Green (SO/FG) staining. In the sham group, the cartilage surface of the knee joint appeared smooth and was devoided of structural damage. At the 2 weeks following DMM, the cartilage surface of the medial tibia exhibited localised roughness, accompanied by a notable reduction in proteoglycans. Additionally, the cartilage adjacent to the synovium hyperplasia emerged, along with the occurrence of osteophytes. At 8 weeks postoperatively, severe localised damage occurred to the medial tibial plateau cartilage with osteophytes at the cartilage margins, perforation of the subchondral bone plate, and resorption of the subchondral bone leading to cystic changes (Figure 1A). As assessed by the modified Mankin score, both the 2‐week and 8‐week scores were significantly higher compared to those of the sham group. The 2‐week scores ranged from 4.0 to 8.3, indicating mild‐to‐moderate OA, whereas the 8‐week scores ranged from 9.3 to 13.0, representing moderate‐to‐severe OA (Figure 1B). Combined with SO/FG staining at 2 and 8 weeks after DMM surgery, the central area of cartilage lesions was located approximately at 1/4–1/2 of the medial tibial plateau cartilage near the synovium (Figure 1C).

To analyse the expression of Piezo1 in distinct regions of the cartilage, we partitioned the weight‐bearing medial tibial plateau into three equal‐width zones as previously reported [27]. The outer zone (adjacent to synovium) had partial cartilage damage with thickened cartilage and osteophytes. The middle zone (weight‐bearing region) had the most severe cartilage damage, and the inner zone (proximal to the central ligament) had the least cartilage lesions (Figure 1D). Immunofluorescence analysis revealed significant reductions in cartilage extracellular matrix COL2 and ACAN in the area of cartilage lesion at 2 and 8 weeks after DMM compared with the sham group (Figure S1A,B). There was no substantial elevation in PIEZO1 level within the 3 regions of the medial tibial plateau cartilage 2 weeks after DMM. In contrast, 8 weeks after DMM, the percentage of PIEZO1‐positive cells markedly elevated in both middle and outer zones, whereas there was no significant change in the inner zone (Figure 1E,F).

### 3.2 Excessive Mechanical Stress Increased Piezo1 Leading to Diminished Chondrocyte Anabolism

**FIGURE 2:**
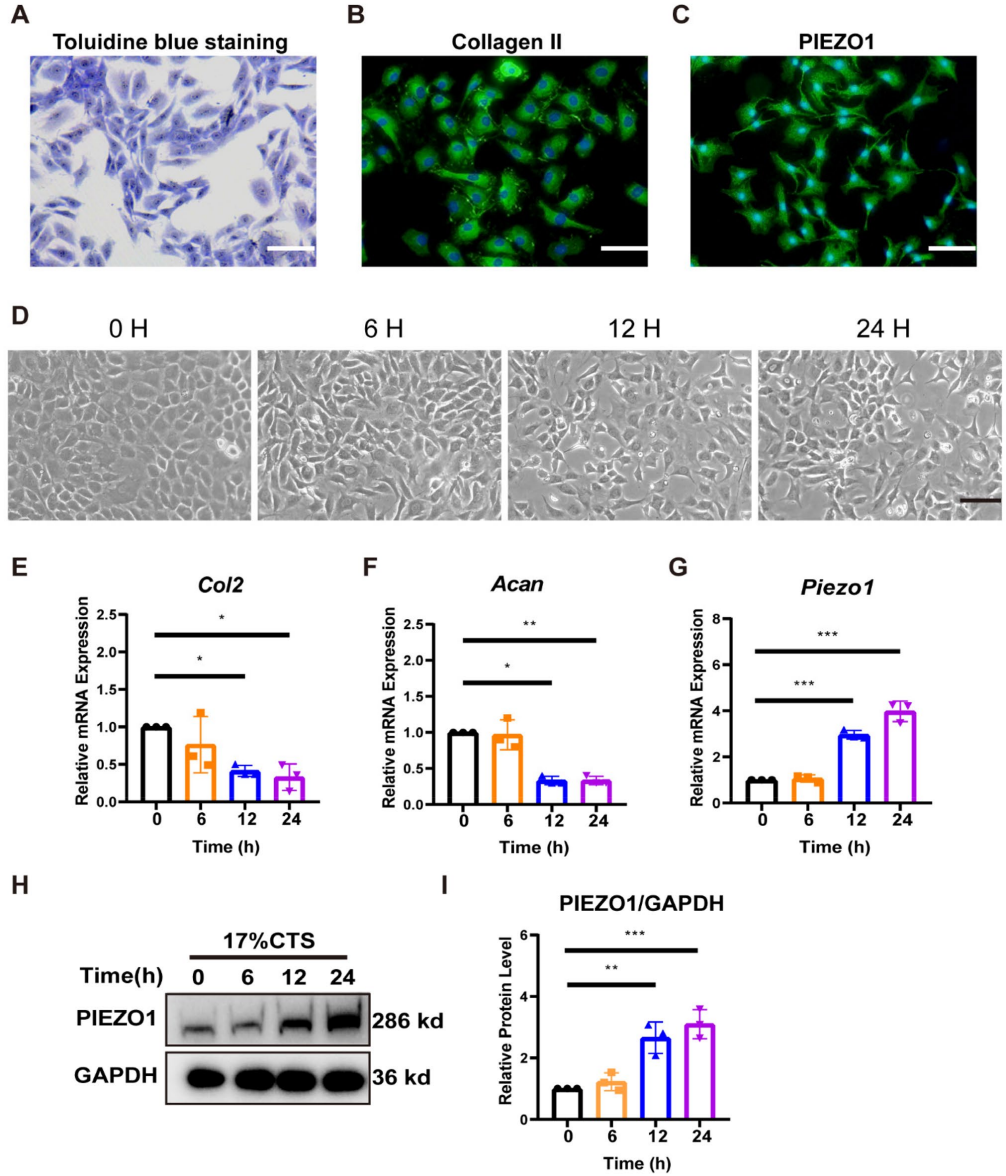
Excessive mechanical stress inhibits chondrocyte anabolism and facilitates the expression of Piezo1. (A–C) Isolated rat primary chondrocytes were stained with toluidine blue and immunofluorescence staining for COL2 and PIEZO1. Scale Bar = 100 μm. (D) Chondrocytes were exposed to cyclic tension stress (CTS) at a magnitude of 17% and a frequency of 0.5 Hz for durations of 0, 6, 12, and 24 h using the Flexcell tension system. Representative images of chondrocyte morphology are shown. Scale bar = 100 μm. (E–G) The mRNA levels of *Col2*, *Acan* and *Piezo1* were quantified through RT‐qPCR analysis (*n* = 3). (H, I) The protein level of PIEZO1 was assessed and quantified via western blotting (*n* = 3). The data were shown as mean ± SD, *p < 0.05, **p < 0.01, ***p < 0.001.

Primary chondrocytes were obtained from 4‐week‐old SD rats and characterised through staining with toluidine blue and COL2 immunofluorescence (Figure 2A,B). Additionally, PIEZO1 exhibited robust expression in the chondrocytes (Figure 2C). Using the Flexcell tension system, cyclic tension stress (CTS, square wave, 0.5 Hz) at a magnitude of 17% was applied to the primary chondrocytes for durations of 0, 6, 12, and 24 h to mimic excessive mechanical stresses experienced by cartilage during OA progression. Chondrocytes in the 0 h group displayed a polygonal shape, whereas CTS treatment for 12 h or 24 h induced cell elongation, filopodia formation, increased intercellular gaps, and vacuolation, suggesting potential cytoskeletal disruption or cell death under prolonged high‐intensity CTS (Figure 2D). After exposure to CTS loading for 12 h, the mRNA levels of *Col2* and *Acan* were significantly reduced in chondrocytes (Figure 2E,F). Meanwhile, the mRNA and protein levels of Piezo1 were notably elevated (Figure 2G–I).

**FIGURE 3:**
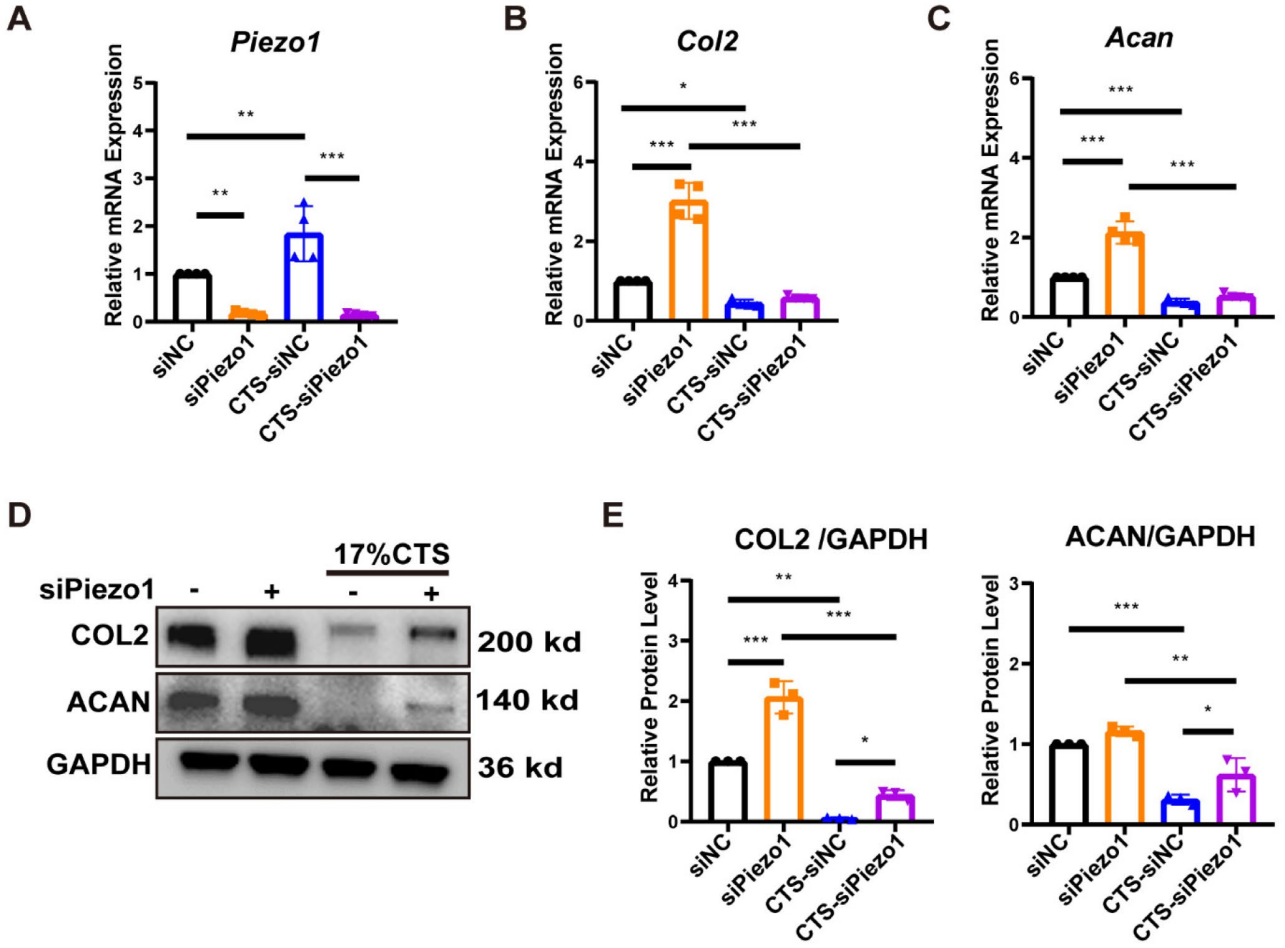
Reducing Piezo1 expression partially alleviates the reduction of COL2 and ACAN caused by excessive mechanical stress. (A–C) After treatment of primary chondrocytes with NC siRNA or *Piezo1* siRNA, 17% CTS (square wave, 0.5 Hz) was applied for 24 h. The mRNA levels of *Piezo1*, *Col2*, and *Acan* were determined by RT‐qPCR (*n* = 4). (D, E) Additionally, western blotting was conducted to determine the protein levels of COL2 and ACAN, and the bands were analysed quantitatively (*n* = 3). The data were expressed as mean ± SD, *p < 0.05, **p < 0.01, ***p < 0.001.

Next, chondrocytes were treated with NC siRNA and *Piezo1* siRNAs, and *Piezo1* siRNA‐3 with the strongest inhibitory effect at both mRNA and protein levels was selected for subsequent experiments (Figure S2A–C). Inhibition of Piezo1 in chondrocytes resulted in a significant increase in the expression of Col2 and Acan. Application of 17% CTS (square wave, 0.5 Hz) for 24 h resulted in increased Piezo1 expression and decreased Col2 and Acan expression in chondrocytes, which was similar to the results in Figure 2 (Figure 3A–C). However, after inhibiting the expression of Piezo1, the reduction of COL2 and ACAN protein levels caused by CTS were significantly alleviated (Figure 3D,E). In summary, Piezo1 mediates the reduction in chondrocyte anabolic metabolism caused by excessive mechanical stress.

### 3.3 Activation of Piezo1 Channels Induced Chondrocyte Homeostatic Imbalance

**FIGURE 4:**
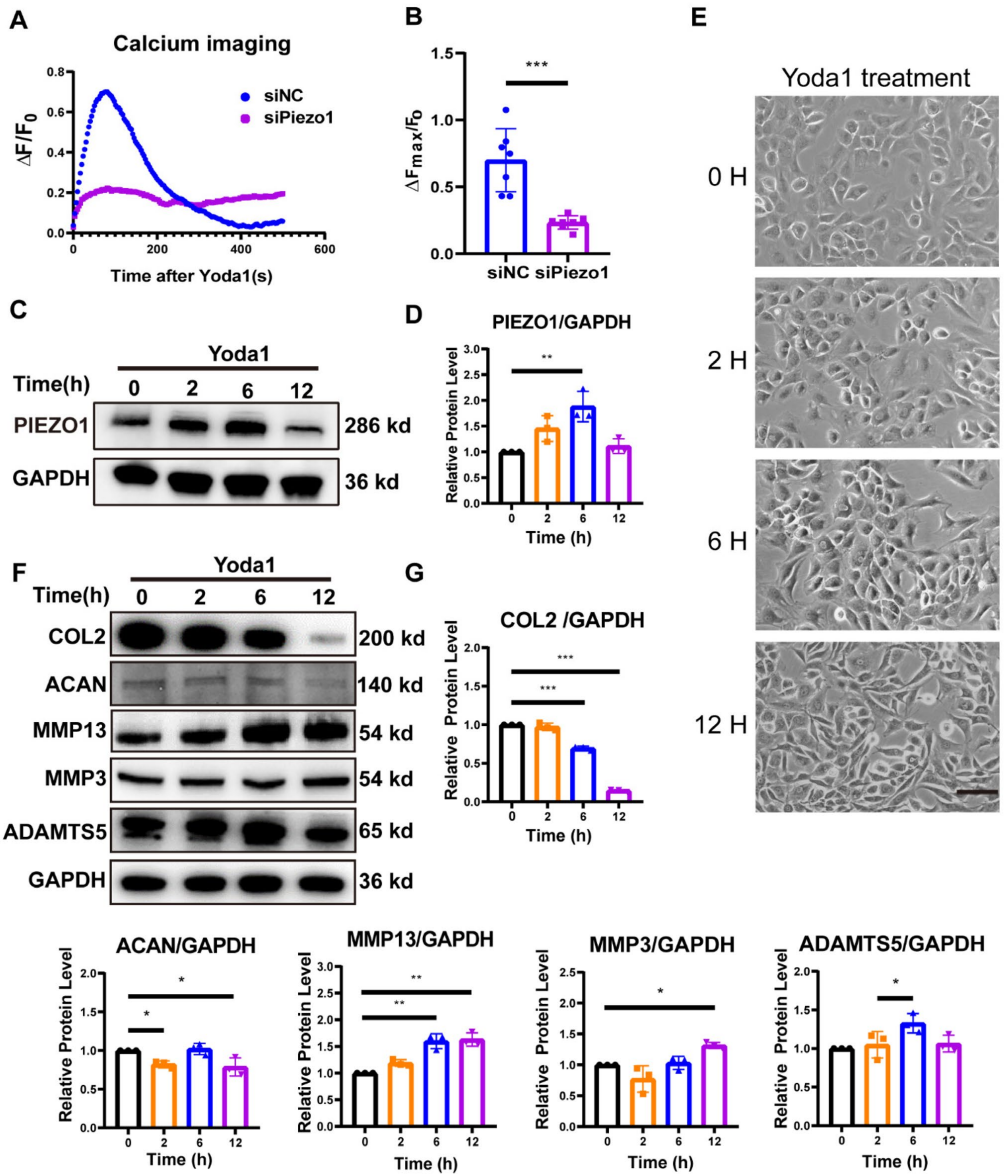
Yoda1 can activate Piezo1 channels, resulting in an imbalance in chondrocyte homeostasis. (A, B) Chondrocytes treated with siNC or si*Piezo1* were incubated with Fura‐8 AM dye, followed by the addition of DMSO or 10 μM Yoda1. Scatter plots demonstrate the change in average intracellular calcium concentration over time. Bar graph analysis compares the maximum change of individual cell calcium concentrations in the siNC and si*Piezo1* groups (*n* = 7). (C, D) After chondrocytes were exposed to 10 μM Yoda1 for 0, 2, 6, and 12 h, PIEZO1 protein levels were analysed by western blot and quantified. (E) Representative morphological images of chondrocytes following treatment with 10 μM Yoda1 for 0, 2, 6 and 12 h. Scale bar = 100 μm. (F, G) Chondrocytes were treated with 10 μM Yoda1 for 0, 2, 6 and 12 h, and protein levels of COL2, ACAN, MMP13, MMP3 and ADAMTS5 were analysed by western blot and quantified (*n* = 3). All data were expressed as mean ± SD, *p < 0.05, **p < 0.01, ***p < 0.001.

To gain further insight into the function of Piezo1 in chondrocytes, we utilised Yoda1, a specific agonist of Piezo1. Calcium imaging showed that 10 μM Yoda1 caused a rapid increase in calcium ion level in chondrocytes, which peaked within 100 s and then gradually declined, creating a calcium transient. In contrast, the calcium signal in *Piezo1* siRNA‐treated chondrocytes was significantly suppressed (Figure 4A,B). Moreover, PIEZO1 protein levels were significantly increased at 6 h after Yoda1 treatment (Figure 4C,D). The above data suggest that Yoda1 can activate Piezo1 channels in chondrocytes to cause calcium inward flow and also induce the expression of PIEZO1. The cell morphology images revealed that after 12 h of Yoda1 treatment, some chondrocytes exhibited altered morphology, appearing spindle‐shaped with filopodia (Figure 4E). Although the anabolic molecules COL2 and ACAN decreased over time in chondrocytes after 0, 2, 6, and 12 h of Yoda1 addition, the catabolic enzymes MMP13, MMP3 and ADAMTS5 were significantly elevated (Figure 4F,G).

### 3.4 Piezo1 Regulated Chondrocyte Homeostasis Through the PI3K/AKT/mTORC1 Signalling Pathway

**FIGURE 5:**
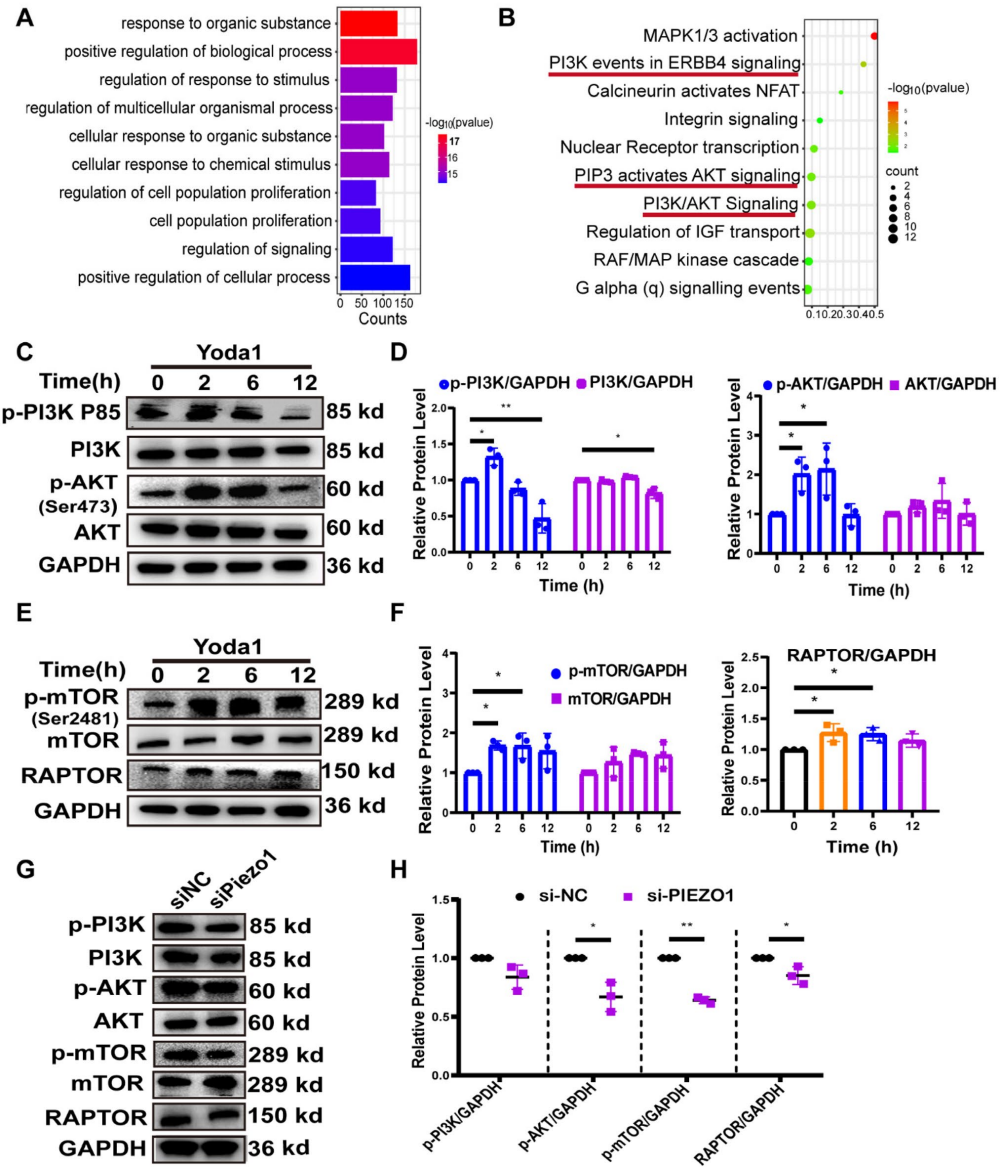
Activation of the Piezo1 channel induces the PI3K/AKT/mTORC1 pathway. (A) RNA sequencing was performed on chondrocytes treated with DMSO or 10 μM Yoda1 for 2 h, and comparative analysis of the data was conducted (*n* = 3). The bar graph illustrates the most significantly up‐regulated biological processes in the GO functional enrichment analysis. (B) Bubble plots demonstrating significantly altered signalling pathways in Reactome enrichment analysis. (C–F) After treatment of chondrocytes with 10 μM Yoda1 for 0, 2, 6 and 12 h, western blot analysis was used to assess expression of p‐PI3K, PI3K, p‐AKT, AKT, p‐mTOR, mTOR and RAPTOR, and protein bands were quantified (*n* = 3). (G, H) The levels of p‐PI3K, PI3K, p‐AKT, AKT, p‐mTOR, mTOR and RAPTOR expression were analysed by western blot after siNC and si*Piezo1*‐treated chondrocytes (*n* = 3). All data were presented as mean ± SD. *p < 0.05, **p < 0.01.

To explore the downstream pathways associated with the mechanosensitive ion channel Piezo1, primary rat chondrocytes were exposed to either DMSO or Yoda1 for 2 h and subjected to RNA sequencing analysis. GO functional analysis of differentially expressed genes (DEGs, |log2FC| ≥ 1, *p* ≤ 0.05) showed that cellular processes (BP) such as cellular self‐defence response, response to external stimuli and cellular signalling regulation were up‐regulated (Figure 5A). Reactome pathway enrichment analysis demonstrated that the PI3K/AKT signalling pathway was significantly enriched after Yoda1 treatment (Figure 5B). Subsequently, western blot analysis showed that protein levels of p‐PI3K and p‐AKT significantly increased at 2 h in chondrocytes treated with Yoda1, peaked at 6 h, and declined at 12 h (Figure 5C,D). In addition, we detected the activation of mTORC1, a key downstream molecule in the PI3K/AKT pathway. The protein levels of p‐mTOR, mTOR and RAPTOR, a specific member of the mTORC1 complex, were significantly elevated and subsequently decreased, consistent with the trend observed for p‐PI3K and p‐AKT (Figure 5E,F). In contrast, the protein levels of p‐AKT, p‐mTOR, and RAPTOR were markedly reduced after inhibition of Piezo1 expression in chondrocytes (Figure 5G,H).

**FIGURE 6:**
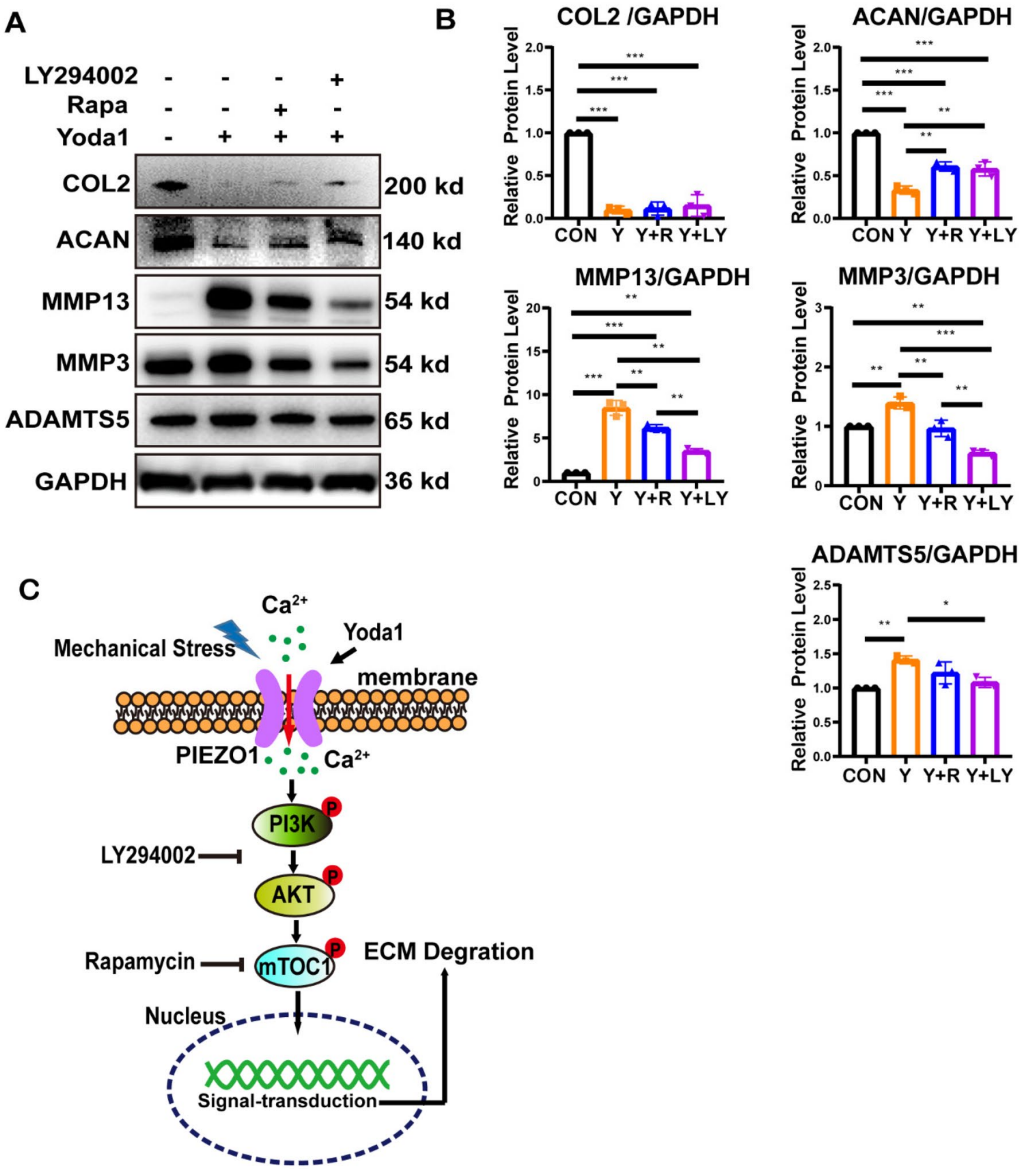
Piezo1 regulates chondrocyte homeostasis through the PI3K/AKT/mTORC1 pathway. (A, B) Chondrocytes were pretreated with LY294002 or rapamycin for 2 h, followed by stimulation with Yoda1 for 12 h. Protein levels of COL2, ACAN, MMP13, MMP3 and ADAMTS5 were analysed by western blotting and protein bands were quantified (*n* = 3). (C) A schematic diagram illustrating the Piezo1‐mediated PI3K/AKT/mTORC1 signalling pathway. All data were presented as mean ± SD. *p < 0.05, **p < 0.01, ***p < 0.001.

To further elucidate the effects of these pathways on chondrocyte homeostasis, compounds LY294002 (PI3K/AKT pathway inhibitor) and rapamycin (mTORC1 inhibitor) were selected for subsequent studies. Chondrocytes were preincubated with LY294002 or rapamycin for 2 h, respectively, and then exposed to Yoda1 for 12 h. Western blot analysis revealed that inhibition of PI3K/AKT or mTORC1 alleviated the Yoda1‐induced reductions in ACAN and increases in MMP13, MMP3 and ADAMTS5 to varying degrees (Figure 6A,B). Furthermore, we validated the efficacy of the inhibitors. Compared to the Yoda1 group, LY294002 significantly reduced p‐AKT and p‐mTOR levels, whereas rapamycin markedly decreased p‐mTOR levels but increased p‐AKT expression (Figure S3A). In conclusion, we have depicted a schematic diagram illustrating the signalling pathway through which Piezo1 in chondrocytes responds to mechanical stress and regulates chondrocyte homeostasis via the PI3K/AKT/mTORC1 pathway (Figure 6C).

## 4 Discussion

OA is a complicated disease with no effective treatment currently available. Understanding the pathological mechanism and key pathways of OA occurrence and progression is crucial for the development of targeted drugs [28]. Our results show that Piezo1, a mechanosensitive calcium channel, is critical in OA development under mechanical stress. The expression of PIEZO1 was significantly elevated in cartilage lesion areas in DMM surgery‐induced OA rats. Piezo1 in chondrocytes responds to excessive mechanical stress and regulates chondrocyte homeostasis by inducing the PI3K/AKT/mTORC1 signalling pathway. Our study delineates Piezo1 spatial activation pattern in mechanically stressed cartilage and uncovers the PI3K/AKT/mTORC1 axis as a key downstream mediator, providing a novel mechanistic link between mechanical overload and OA progression.

It is widely believed that normal physiological loading maintains cartilage structure and homeostasis, whereas prolonged excessive mechanical loading can lead to cartilage degeneration and damage, ultimately contributing to the onset and progression of OA, but many underlying mechanisms remain unclear [29, 30]. In recent years, several studies have indicated the significant role of the mechanosensitive ion channel Piezo1 in the mechanotransduction of articular cartilage, where it can sense high‐intensity mechanical strains and promote the progression of OA [31, 32, 33]. Recently published studies have also shown that selectively inactivating Piezo1 in chondrocytes attenuates the pathological process of OA [34]. However, different viewpoints have also been proposed. Rho et al. suggested that the expression level of the *Piezo1* gene is not affected in a rat model of sodium iodoacetate‐induced knee OA [35]. Young et al. demonstrated through *Gdf5*‐cre conditional knockout mouse models that Piezo1/2 has limited contributions to the progression of OA [36]. Therefore, the role of Piezo1 in OA and its molecular mechanism need to be further studied.

DMM surgery of the knee joint can cause an abnormal increase of mechanical load on the medial tibial plateau cartilage, resulting in continuous cartilage degeneration, and then the development of OA. It is considered an ideal model to simulate OA caused by excessive mechanical [37, 38]. In this study, the medial tibial plateau cartilage of rats after DMM surgery was divided into three equal zones. The cartilage lesions were severe in the outer 1/3 region and middle 1/3 region (weight‐bearing region) close to the synovium, whereas the lesions were milder in the inner 1/3 region, which is similar to previous reports [39]. At 8 weeks after DMM, PIEZO1 expression on medial tibial cartilage showed regional differences. PIEZO1 expression levels were significantly elevated in the outer 1/3 and middle 1/3, whereas there was no significant change in the inner 1/3, suggesting that the up‐regulation of PIEZO1 is associated with cartilage damage and osteophyte formation due to abnormal mechanical forces. In vitro studies have also shown that Piezo1 responds to excessive mechanical stress, leading to homeostatic imbalances in chondrocytes. Interestingly, reduction of Piezo1 expression by siRNA significantly increased Col2 and Acan levels in chondrocytes, indicating a close link between Piezo1 and cartilage matrix synthesis.

To identify Piezo1‐related downstream molecules, we performed RNA sequencing analyses and screened for the PI3K/AKT pathway. Further results showed that Piezo1 regulates the activity of PI3K/AKT/mTORC1. This pathway is closely linked to autophagy, senescence, and apoptosis in chondrocytes [40]. However, how Piezo1 specifically regulates OA progression through the PI3K/AKT/mTORC1 pathway requires further investigation. Given the pathway's complexity and its pleiotropic functions in chondrocytes (e.g., metabolic regulation, inflammatory response and cell survival), we further analysed the impact of Piezo1 activation on the mTORC1 complex. The mTORC1 complex is a key downstream target of PI3K/AKT and consists of three core components, mTOR, Raptor and mLST8. Of these, Raptor is a specific member and regulates the activity of mTORC1 [41]. We determined the regulatory role of Piezo1 on mTORC1 by assaying p‐mTOR and Raptor. Further, chondrocyte homeostasis dysregulation caused by Yoda1 was alleviated after rapamycin. Notably, rapamycin inhibited p‐mTOR, while increasing p‐AKT expression, which may be due to negative feedback regulation [42]. In addition, LY294002 showed stronger relief than rapamycin after dual inhibition of p‐AKT and p‐mTOR, which not only confirms the role of the Piezo1‐PI3K/AKT/mTORC1 signalling axis, but also suggests that this pathway may contribute to OA progression via pleiotropic mechanisms (e.g., modulating autophagy or crosstalk with inflammatory pathways such as NF‐κB). Future studies should further elucidate the precise regulatory network of the Piezo1‐PI3K/AKT/mTORC1 axis in chondrocytes and its pathological contributions to OA.

In our bioinformatics data, activating Piezo1 led to significant changes in a number of molecular pathways associated with inflammation, including NF‐κB, IL‐17 and cytokine‐cytokine receptor interaction pathways (Figure S3B). Our results demonstrate that treatment with 10 μM Yoda1 for 2 h promoted NF‐κB nuclear translocation and increased phospho‐NF‐κB levels (Figure S3C,D), which aligns with our RNA‐seq findings. Thus, Piezo1 promotes inflammation in OA, as previously reported [43]. However, the regulatory role of Piezo1‐NFκB in chondrocyte metabolism and its crosstalk with the PI3K/AKT/mTORC1 pathway require further investigation. It should be added that Piezo1 is also involved in the infrapatellar fat pad (IFP) and synovial membrane (SM) of OA. Within the IFP‐SM anatomo‐functional unit (AFU), Piezo1 may mediate interactions between immune cells and vascular endothelial cells, promoting the formation of an inflammatory microenvironment and transmission of pain signals [44]. In addition, we observed a marked enrichment of osteoclast differentiation pathways, and chondrocyte‐osteoclast crosstalk plays an important role in OA [45]. It would be interesting to study the effect of Piezo1 on the cross‐linking between cartilage and subchondral bone in OA.

Our study has several limitations that need to be addressed. Considering the lack of highly specific Piezo1 inhibitors, the direct effects of the Piezo1‐PI3K/AKT/mTORC1 pathway on OA were not further validated in rats in this study. Although GsMTx4 has been reported to attenuate abnormal mechanical stress‐induced OA in vivo [21], this cationic mechanosensitive channel inhibitor non‐specifically targets both Piezo and other channels (e.g., TRP family) [46]. In future studies, we anticipate using transgenic mice or AAV targeting strategies to determine the effects of Piezo1 and its downstream pathways on the OA process. Additionally, although we have demonstrated the crucial role of the Piezo1‐PI3K/AKT/mTORC1 signalling pathway in chondrocyte homeostasis, the downstream mechanisms by which Piezo1 regulates this pathway and the role of inflammatory signalling (e.g., NF‐κB) within it still require further investigation.

In different types of cells, Piezo1 exerts distinct functions depending on the type and intensity of mechanical stress [47]. For example, Piezo1 in osteoblast‐lineage cells is essential for the normal development and growth of the skeleton, whereas Piezo1 in chondrocytes may promote the development of OA [32]. Since Piezo1 has complex functions, more studies are needed to understand its role in synovial joints. Such knowledge could facilitate the development of targeted therapies for OA.

## 5 Conclusion

In conclusion, our study shows that the up‐regulation of Piezo1 in the cartilage of DMM‐induced OA rats is correlated with cartilage damage and osteophyte formation due to abnormal mechanical load. Excessive mechanical stress or Yoda1 activates Piezo1, which inhibits chondrocyte anabolism and promotes catabolism via the PI3K/AKT/mTORC1 pathway. Piezo1 and its downstream signalling pathways may be potential targets for preventing or treating OA.

## Author Contributions

**Yuanyuan Han:** data curation (equal), investigation (equal), methodology (equal), validation (equal), writing – original draft (equal). **Fangyan Cheng:** formal analysis (equal), methodology (equal), visualization (equal), writing – original draft (equal). **Xinyan Li:** data curation (equal), methodology (equal), validation (equal), writing – original draft (equal). **Jiahao Yu:** data curation (equal), methodology (equal), software (equal), validation (equal). **Guimiao Li:** methodology (equal), validation (equal), visualization (equal). **Wei Chen:** conceptualization (equal), investigation (equal), supervision (equal). **Jiaxue Zhang:** data curation (equal), methodology (equal), visualization (equal). **Chen Feng:** conceptualization (equal), investigation (equal), supervision (equal), writing – review and editing (equal). **Yingze Zhang:** conceptualization (equal), investigation (equal), project administration (equal), supervision (equal). **Juan Wang:** conceptualization (equal), funding acquisition (equal), project administration (equal), writing – review and editing (equal).

## Ethics Statement

The animal study protocol was reviewed and approved by the Laboratory Animal Ethics and Welfare Committee of Hebei Medical University (Approved number: IACUC‐Hebmu‐2022044).

## Conflicts of Interest

The authors declare no conflicts of interest.

## Supporting information


Data S1.

## Data Availability

The data that support the findings of this study are available from the corresponding author upon reasonable request.
