# Supplementary material for: The Mechanosensitive Ion Channel Piezo1 Regulates Chondrocyte Homeostasis Through the PI3K/AKT/mTORC1 Pathway in Osteoarthritis
Source: J Cell Mol Med. 2025 Jul 31;29(15):e70734. doi: 10.1111/jcmm.70734 (PMC12313542; doi:10.1111/jcmm.70734)
Supplement: Supplementary file 1 — Data S1. [file JCMM-29-e70734-s001.docx]

Supplementary Material

# Supplementary Figures and Tables

## Supplementary Figures


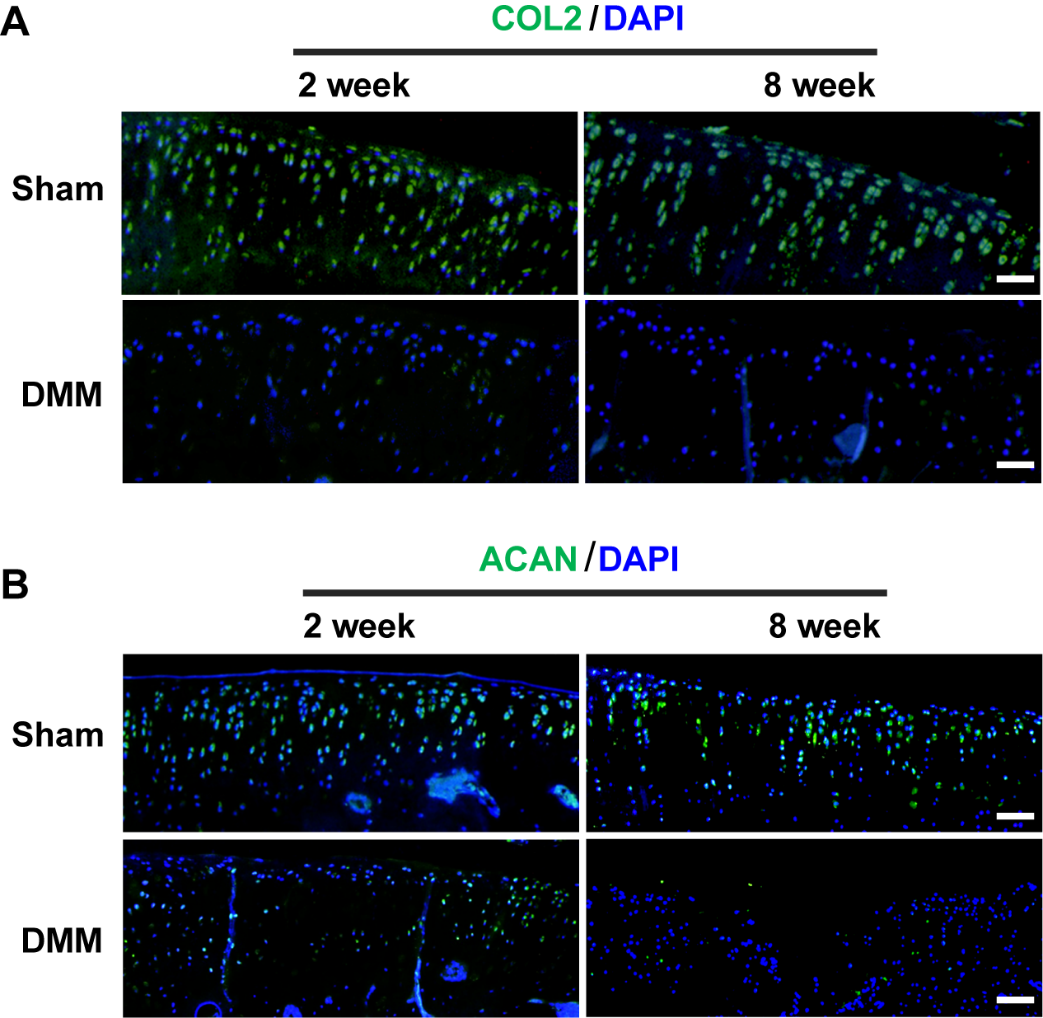


**Supplementary Figure S1.** The expression levels of both COL2 and ACAN were reduced in the articular cartilage of DMM-induced OA rats. **(A)** The immunofluorescence staining for COL2 in cartilage lesion region at 2 and 8 weeks after sham or DMM surgery. **Scale Bar, 100 μm** **(B)** The immunofluorescence staining for ACAN in cartilage lesion region at 2 and 8 weeks after sham or DMM surgery. Scale Bar, 100 μm


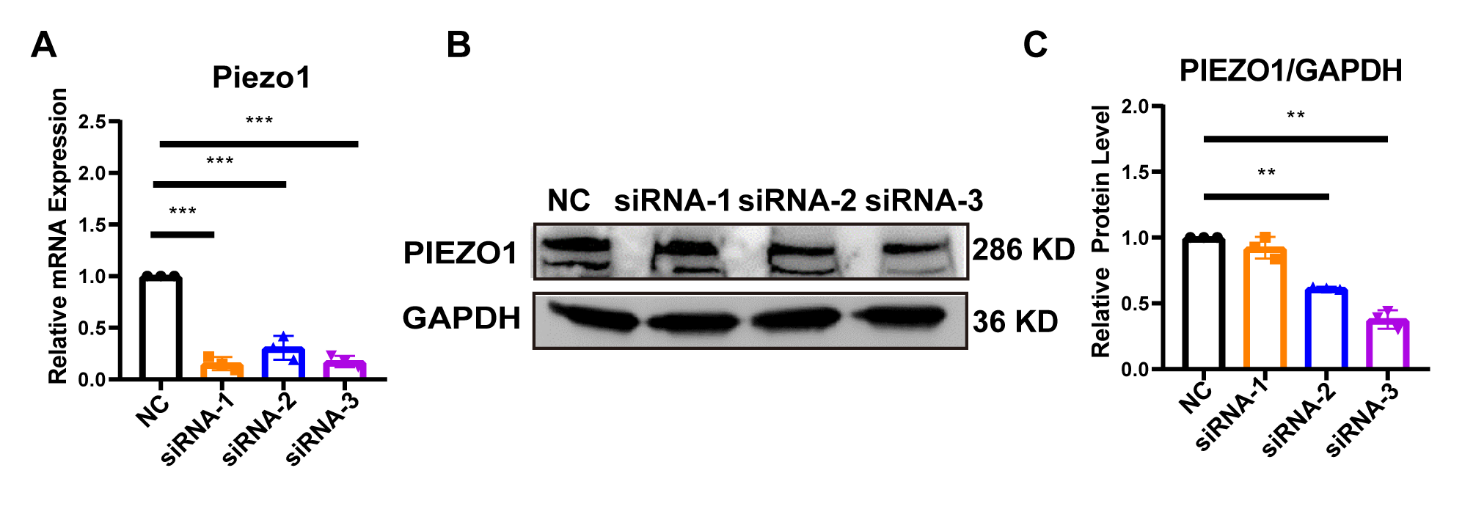


**Supplementary Figure S2.** Piezo1 siRNA treatment reduced the expression level of Piezo1 in chondrocytes. **(A)** Relative expression levels of Piezo1 mRNA were analyzed by RT-qPCR after 48 h of treatment of chondrocytes with NC-siRNA, Piezo1 siRNA-1, Piezo1 siRNA-2 and Piezo1 siRNA-3 (n=3). **(B, C)** Western blot analyzed the protein levels of PIEZO1 and quantified the bands (n=3). The data are expressed as mean ± SD, **P < 0.05, **P < 0.01, ***P < 0.001*


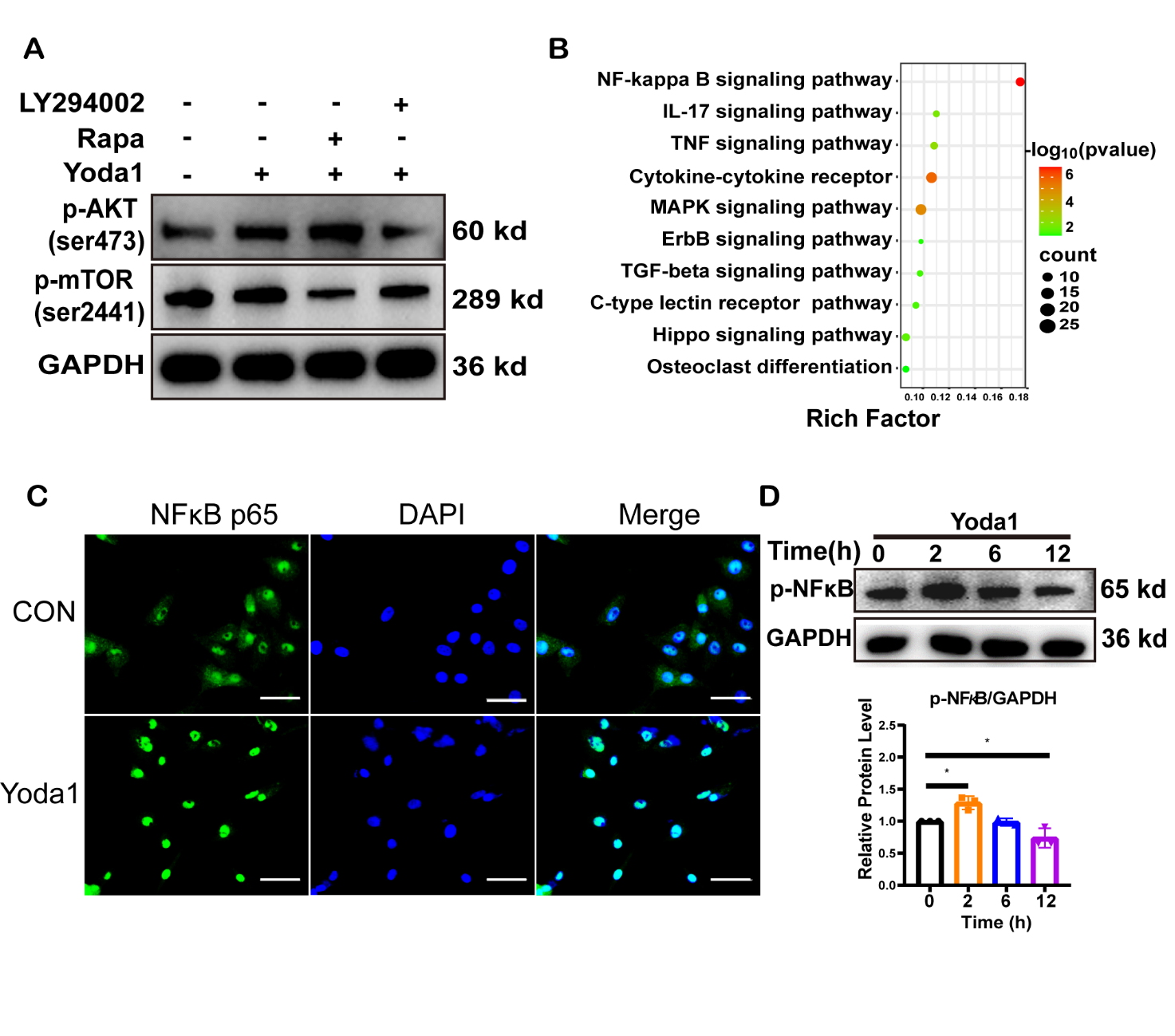


**Supplementary Figure S3.** Yoda1 can induced the NFκB signaling pathway. (A) Chondrocytes were preincubated with LY294002 or rapamycin for 2 h, respectively, and then exposed to Yoda1 for 6 h. Western blot was performed to detect the protein levels of p-AKT and p-mTOR. (B) Chondrocytes were treated with DMSO or Yoda1 for 2 h followed by RNA sequencing, and the data were analyzed comparatively compared to the DMSO group (n=3). KEGG enrichment analysis bubble plot showing significantly enriched upregulated signaling pathways. (C) Representative images of cellular immunofluorescence of NFκB p65 after 10 μM Yoda1 treatment of chondrocytes for 2 hours. (D) Western blot analysis and quantification of protein levels of p-NFκB after 10 μM Yoda1 treatment of chondrocytes for 0, 2, 6 and 12 hours. The data are expressed as mean ± SD, **P < 0.05*

## Supplementary Tables

**Supplementary Table S1** The sequences of Piezo1 siRNAs.

| siRNAs | Sense (5’-3’) | Antisense (5’-3’) |
| --- | --- | --- |
| siRNA-1 | GCUUCUACCUGCUGCUCUUTT | AAGAGCAGCAGGUAGAAGCTT |
| siRNA-2 | CCCAAGAGCUUCUAGCCAATT | UUGGCUAGAAGCUCUUGGGTT |
| siRNA-3 | GCCCAUUGAUGUCACUGUUTT | AACAGUGACAUCAAUGGGCTT |
